# Reducing Haemorrhagic Transformation after Thrombolysis for Stroke: A Strategy Utilising Minocycline

**DOI:** 10.1155/2013/362961

**Published:** 2013-04-04

**Authors:** David J. Blacker, David Prentice, Anthony Alvaro, Timothy R. Bates, Michael Bynevelt, Andrew Kelly, Lay Kun Kho, Edith Kohler, Graeme J. Hankey, Andrew Thompson, Taryn Major

**Affiliations:** ^1^Department of Neurology and Clinical Neurophysiology, Sir Charles Gairdner Hospital, Nedlands, Western Australia and School of Medicine and Pharmacology, University of Western Australia, Nedlands, WA 6009, Australia; ^2^Department of General Medicine, Royal Perth Hospital, Perth, WA 6000, Australia; ^3^Department of Neurology, Fremantle Hospital, Fremantle, WA 6160, Australia; ^4^Stroke Unit, Swan District Hospital, Middle Swan WA 6056, School of Medicine and Pharmacology, University of Western Australia, Nedlands, WA 6009, Australia; ^5^Department of Neurological Intervention and Imaging Service of Western Australia, Sir Charles Gairdner Hospital, Nedlands, WA 6009, Australia; ^6^Stroke Unit, Swan District Hospital, Middle Swan, WA 6056, Australia; ^7^Department of Neurology, Royal Perth Hospital, Perth WA 6000, School of Medicine and Pharmacology, The University of Western Australia, Nedlands, WA 6009, Australia; ^8^Data Analysis Australia, Nedlands, WA 6009, Australia

## Abstract

Haemorrhagic transformation (HT) of recently ischaemic brain is a feared complication of thrombolytic therapy that may be caused or compounded by ischaemia-induced activation of matrix metalloproteinases (MMPs). The tetracycline antibiotic minocycline inhibits matrix MMPs and reduces macroscopic HT in rodents with stroke treated with tissue plasminogen activator (tPA). The West Australian Intravenous Minocycline and TPA Stroke Study (WAIMATSS) aims to determine the safety and efficacy of adding minocycline to tPA in acute ischaemic stroke. The WAIMATSS is a multicentre, prospective, and randomised pilot study of intravenous minocycline, 200 mg 12 hourly for 5 doses, compared with standard care, in patients with ischaemic stroke treated with intravenous tPA. The primary endpoint is HT diagnosed by brain CT and MRI. Secondary endpoints include clinical outcome measures. Some illustrative cases from the early recruitment phase of this study will be presented, and future perspectives will be discussed.

## 1. Introduction

Haememorrhagic transformation (HT) of recently infarcted brain causing intracerebral haemorrhage (ICH) is a feared complication of ischaemic stroke that is clearly increased with thrombolytic and anticoagulant medications [1]. It should be noted that ICH can occur spontaneously, typically at the core of an ischaemic infarction, likely related to breakdown of the blood brain barrier (BBB). Activation of proteins such as the metalloproteinases (MMPs) by the ischaemic cascade is likely one of the elements in BBB leakage [1], resulting in haemorrhagic transformation and increased oedema. Various studies of thrombolysis have demonstrated increased rates of ICH compared with placebo, ranging from 1.7% [2] to 8.8% [3], with some of the differences accounted by different definitions of ICH. In the pivotal NINDS study [4], ICH occurred in 6.4% of tPA treated patients (compared with 0.6% in the placebo group), and mortality was 47%. There appears to be different “forms” of ICH following thrombolysis, ranging from symptomatic parenchymal haematoma with a very high mortality through to asymptomatic minor haemorrhagic transformation which is considered an epiphenomena of reperfusion and has been seen in up to 39.5% of patients in one series [5]. Although it has been suggested [5] that minor haemorrhagic transformation may not have any impact on clinical outcome, some recent observational data [6] suggests that even asymptomatic HT is linked to poor outcome in ischaemic stroke patients.

Any treatment that could reduce the risk of tPA related ICH could substantially reduce mortality, given the high case fatality rate of symptomatic haematoma. Such a treatment would thereby improve the risk/benefit ratio for thrombolytic therapy. One potential candidate medication is minocycline, particularly because of its ability to inhibit the expression of matrix metalloproteinases (MMPs). The MMPs are a group of proteins involved in the physiological breakdown of extra-cellular matrix. Animal models of cerebral ischaemia have found elevated levels of the MMPs [7, 8]. TPA may increase the risk of HT by amplifying MMP levels in the setting of ischaemia [9, 10], thereby reducing potential benefits of recanalization. 

Two animal studies [11, 12] combining minocycline with tPA in rodent models of ischaemic stroke have demonstrated significant reductions in MMP-9 levels and shown as much as a twofold reduction in ICH compared with placebo. Human stroke studies have shown safety and tolerability of orally [13] and intravenously (IV) [14] administered minocycline preparations. There is also safety and pharmacokinetic data on the combination of minocycline and tPA in animal [12] and human studies [14]. There have been three randomised trials of minocycline in human stroke. An open label study [13] of oral minocycline administered a mean of 12.4 hours after stroke onset showed promising results with improvement in NIHSS being seen as early as one week. A further similar study was also positive [15]. The two studies did not include patients treated with tPA. Our group has recently completed a pilot study [16], The Perth Intravenous Minocycline Stroke Study (PIMSS), of IV minocycline in ischaemic and haemorrhagic stroke, up to 24 hours after symptom onset, with a mean time to treatment of 10.6 hours. This study was neutral but provided further safety data and included a small number of patients concurrently treated with tPA. We are presently conducting a meta-analysis of these three small and somewhat heterogeneous studies. PIMSS [16] included 14 subjects treated with tPA, 8 of whom received minocycline and 6 standard care; there was one subject with haemorrhagic transformation in each group. The Minocycline to Improve Neurological Outcome in Stroke (MINOS) trial [14] was a dose-finding study of 60 subjects with ischaemic stroke assigned to receive 3, 4.5, 6, or 10 mg/kg of intravenous minocycline daily for 72 hours, commencing within 6 hours of stroke onset. Thirty-six subjects were also treated with tPA, and there were no cases of severe HT reported. These limited data therefore provide some information regarding the combination of IV minocycline and tPA in human subjects with acute ischaemic stroke. 

The West Australian Intravenous Minocycline and TPA Stroke Study (WAIMATSS) [17] is a multicentre, prospective, and randomised pilot study of intravenous minocycline, 200 mg 12 hourly for 5 doses, compared with standard care, in patients with ischaemic stroke treated with intravenous tissue plasminogen activator (tPA). The first dose will be administered within 6 hours of stroke onset. The objectives of WAIMATSS are the following.

1. To test the hypothesis that subjects with acute stroke treated with IV minocycline and tPA have fewer intracranial haemorrhages (ICH) compared with those treated with IV tPA and standard care, seen on routine followup CT scans, 24 ± 8 hours posttreatment.
2. As a substudy, to test the hypothesis that subjects with acute stroke treated with IV minocycline and tPA have fewer intracranial haemorrhages (ICH) compared with those treated with IV tPA and standard care, seen on MRI scans performed on day 5–7 posttreatment.
3. To determine the magnitude of this effect, with a view to estimating the sample size required for a phase III study.
4. To determine the feasibility of a phase III study, based on this pilot study design and preliminary data.
5. To test the hypothesis that subjects treated with both IV minocycline and IV tissue plasminogen activator (tPA) have improved clinical outcomes compared with those treated with IV tPA and standard care.

## 2. Materials and Methods

### 2.1. Setting

Emergency departments of the four metropolitan teaching hospitals with stroke units in Perth, Western Australia.

### 2.2. Design

A prospective randomised open label blinded endpoint evaluation (PROBE) pilot trial. 

### 2.3. Study Population

*Inclusion Criteria*

Subjects must meet the standard inclusion criteria for use of intravenous tPA, be at least 18 years of age, and provide informed consent. 

IV tPA to be administered within 4.5 hours of stroke onset.

Trial intervention can be administered within 6 hours of stroke onset.

*Exclusion Criteria*

Standard exclusion criteria are routine use of tPA, as per local and national guidelines, and patients treated with both tPA and thrombectomy or another endovascular technique. 

*Specific Exclusion Criteria for the Trial*

1. Evidence of other significant CNS diseases that interfere with assessment (e.g., tumor, multiple sclerosis).
2. Known allergy to tetracyclines/intolerance of minocycline.
3. Known systemic lupus erythematosus.
4. Idiopathic intracranial hypertension.
5. Concurrent treatment with vitamin A or retinoids.
6. Participation in another clinical drug trial.
7. Known significant renal failure, CLcr <30 mL/min by the Cockcroft-Gault equation.
8. Known significantly abnormal liver function tests (ALT > ×3 ULN).
9. Known thrombocytopaenia <100 × 10^9^/L.
10. Concurrent infection requiring antibiotic treatment.
11. Pregnancy.
12. Severe stroke or other comorbidities likely to result in the patient dying within a week.

### 2.4. Baseline Measures

The baseline clinical assessment is part of routine stroke unit care and comprises a complete medical history, physical examination including vital signs, and NIHSS.

### 2.5. Randomisation

Once consent is obtained, a sealed envelope containing treatment allocation to either IV minocycline or standard care (i.e., no minocycline) will be opened. These sealed envelopes will be kept with the “Stroke kit” that is used at each of the teaching hospitals and used by clinicians from the stroke units. Batches of envelopes with equal numbers of randomly assigned treatment allocation will be provided to each study site. This procedure may be upgraded to a web-based randomisation system in the near future.

### 2.6. Intervention

Following randomisation patients will be treated with either intravenous minocycline 200 mg then 12 hourly for a total of 5 doses, that is, for 48 hours, or no minocycline. All participants will receive routine stroke unit care. The minocycline is to be commenced as soon as possible, ideally whilst the tPA infusion is still running but no later than 6 hours after stroke onset. The dose has been selected to approximate the “mid-range” of a recent dose-finding study [14] of IV minocycline in human acute stroke patients.

### 2.7. Primary Outcome

The primary outcome measure is the presence of any ICH on the routine followup CT brain scan, performed 24 ± 8 hours posttreatment with tPA. This has been selected as an intentionally broad endpoint, to maximise the detection of events. Standard baseline CT brain will be performed as per usual practice in the assessment of acute stroke patients. As per usual practice, a followup noncontrast CT is performed on the second day; for the purpose of the study, the timing of this scan will be standardised to occur 24 hours ± 8 hours after the onset of the tPA infusion. Two neuroradiologists blinded to treatment allocation will independently review all pairs of CT scans and classify the presence of any ICH and ICH according to the European Cooperative Acute Stroke Study (ECASS) [3] criteria.

The ECASS Classification is as follows.

 Haemorrhagic infarction type 1 (HI-1): small petechiae along the margins of the infarct.
 Haemorrhagic infarction type 2 (HI-2): confluent petechiae within the infarcted area but without space occupying effect.
 Parenchymal haematoma type 1 (PI-1): a haematoma in <30% of the infarcted area with some slight space occupying effect.
 Parenchymal haematoma type 2 (PI-2): a dense haematoma >30% of the infarcted area with substantial space occupying effect.
 Additionally, note will be made of any other ICH, defined as any area of increased attenuation consistent with blood, and seen within the intracranial compartment, that was not present on the pretreatment CT. This will account for the rare circumstance where HT is noted outside the infarct area.

### 2.8. Secondary Outcome

1. Symptomatic ICH: any ICH temporally related to deterioration in the patient's condition during the hospital admission.
2. Symptomatic ICH with worsening by 4 or more points on the NIHSS score, during the hospital admission.
3. NIHSS on days one and seven.
4. The modified Rankin score and Barthel index by “blinded” telephone interview at days 30 and 90.

### 2.9. MRI substudy

Subjects will be invited to participate in an MRI substudy in which an MRI with the examination being performed between days 5 and 7 after stroke. The images will be examined by two neuroradiologists blinded to treatment allocation, for signs of ICH. The 1.5T MRI will be used at each hospital site, and a standard stroke protocol will be utilised, also including gradient echo and susceptibility weighted (SWI) sequences, which are more sensitive for the detection of blood products than conventional MRI sequences.

The most sensitive definition of ICH will be any area of focal markedly reduced signal within the infarcted tissue seen on the gradient echo or SWI sequences. This will be used to enable the greatest sensitivity and hence detection of ICH. ICH outside the area of infarct will also be counted.

The ICH will be quantified and recorded in terms of absolute volume (the greatest orthogonal three-dimensional diameter) and relative volume compared with the volume of infarction. In the final report on the study, the plan is to present a montage of all CTs and MRIs with ICH in each group. 

### 2.10. Statistical Analysis and Sample Size Calculations

The study has been designed as a pilot study, with a view to estimating the strength of a possible treatment effect. One animal model [12] demonstrating a twofold reduction in macroscopic haemorrhagic transformation was based on 35 rats treated with tPA and 31 with combined tPA and minocycline. It is estimated that the number of patients recruited to this human study will approximate these figures and could well provide the basis for sample size calculations for a higher phase, definitive study. Using the very “inclusive” definition of “any ICH” should maximise the chance of identifying an effect. An MRI based study [5] found evidence of some haemorrhagic transformation in up to 39.5% of patients treated with tPA up to 6 hours after symptom onset. We thus expect that our MRI substudy may yield a high event rate. ICH rates in the minocycline versus the standard care group will be compared using paired *t*-tests. 

Our primary endpoint might be regarded as a “surrogate” for a treatment response or “proof of principle” rather than necessarily as a meaningful clinical result. This would likely require a much larger study, based upon the rates of symptomatic ICH observed in the large treatment trials of tPA. 

We have made some preliminary calculations of possible sample sizes based on varying rates of haemorrhagic transformation, strength of treatment effect (assuming either 25% or 50% reductions), and varying study power, presented in Table 1.

For example, if the estimated frequency of ICH is 10% and the study drug has a 50% treatment effect, a study with 80% power to show a significant effect would require 286 patients in each of the treatment and nontreatment groups. Whilst such study would not be feasible locally, it could potentially be achieved at a national level with cooperation of stroke centres around Australia and New Zealand. Our pilot human study [17] should provide more reassurance of the magnitude of a treatment effect (and hence guide sample size calculations) than the available animal data.

In the MRI substudy, where the frequency of ICH is up to 40%, 107 patents would be required. The clinical assessments have been chosen to match those used in the Perth Minocycline Stroke Study, to potentially enable pooling of results. 

## 3. Study Duration

Patient recruitment commenced in March 2012 and is planned to continue for approximately 2 years. Approximately 70 patients per year are treated with tPA at these four centres. Allowing for patients who may be unwilling or unable to give consent for participation in a clinical trial, we conservatively estimate that 30 patients per year could potentially be enrolled, resulting in 60 patients over two years. Additional funding may be sought in the future to conduct a higher phase trial.

## 4. Further Study Details

Steering Committee. The steering committee shall meet every 3 months from the commencement of the study and review procedures and any unexpected difficulties with the study. 

Data Monitoring and Safety. A senior local neurologist has agreed to review safety issues and adverse events. He will review all AEs and SAEs and have access to “unblinded” data. He will notify the ethics committee accordingly regarding any safety concerns. 

Trial Registration. The study has been registered with the Australian and New Zealand Clinical Trials Registry. Trial number is ACTRN12611001053910.

## 5. Results and Discussion

Recruitment commenced at the main tertiary site in March 2012, at one peripheral hospital site in October 2012, and screening began at two other sites from December 2012. By early January 2013, twelve subjects had been recruited, nine at the main tertiary site and three from the peripheral hospital.

At the main tertiary site, from March 2012 to December 2012, 32 patients were treated with IV tPA, but 24 were not recruited for WAIMATSS for the following reasons.

- Seven were unable to provide informed consent for a clinical trial.
- Six proceeded to concurrent catheter angiography (five of whom underwent thrombectomy).
- In four cases the investigators were not notified.
- In three cases the patients declined to participate.
- One patient had known preexisting renal failure.
- One patient was allergic to tetracycline antibiotic.
- One patient probably had benign intracranial hypertension.
- In one case, after review by the investigators, the patient was diagnosed as having a conversion disorder and was not invited to participate.

An amendment to the protocol was approved by the local ethics committee at the main tertiary site in November 2012 to allow for the use of informed assent given by the next of kin; it is hoped that this will allow for increased recruitment. The concurrent use of endovascular treatments such as thrombectomy has excluded 25% of tPA treated patients at the main tertiary site. The commencement of screening from December 2012 at two additional sites: one without access to interventional neuroradiology and the other where thrombectomy is used less frequently may result in increasing recruitment. 

Thus far, the practical aspects of the study have run smoothly. There has been one serious adverse event: oro-lingual angioedema in a subject treated with tPA alone and not minocycline. 

Blood products have been detected in two subjects (and the neuroradiologists remain blind to the treatment allocated to these). 

**Figure 1:**
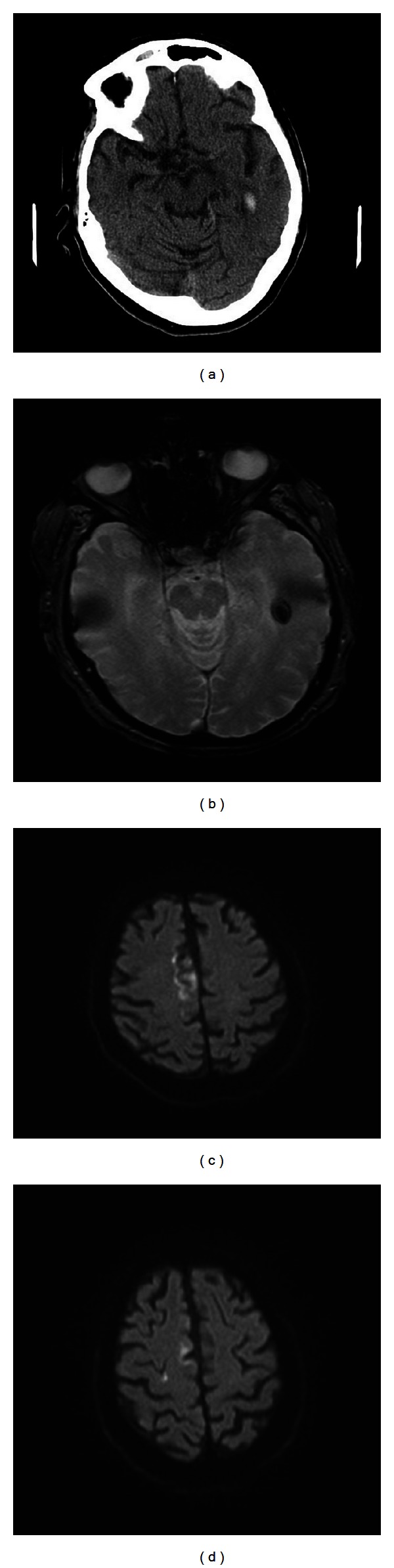
Subject presented with acute left hemiparesis, leg more affected than arm. Routine day one CT (a) and day 5 MRI (b) demonstrate a small haemorrhage in the left temporal lobe, away from the main area of infarction, shown on diffusion weighted MRI (c) and (d), mainly in the territory of the right anterior cerebral artery. No clinical deterioration was detected.

In Figure 1 blood was detected on CT and MRI in a region away from the infarction.

**Figure 2:**
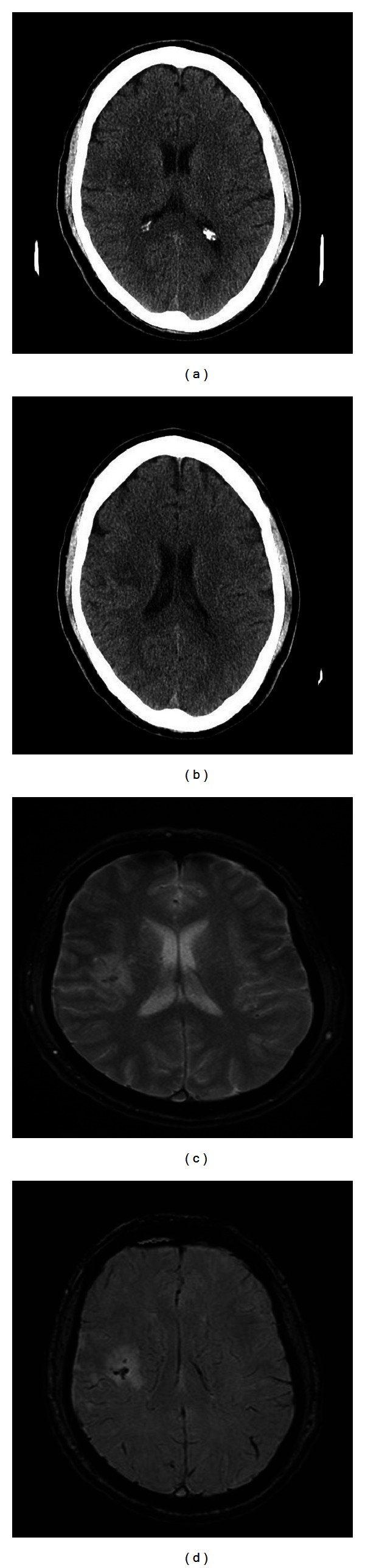
Subject presented with mild left hemiparesis. The day one CT (a) and (b) shows a small infarct in the right subcortical white matter, with a suggestion of increased signal within the central portion. The gradient echo (c) and susceptibility weighted image (d) MRI sequences performed on day seven suggest a small amount of blood within the infarct. No clinical deterioration was detected.

In Figure 2 a small amount of blood was visible only on the MRI sequences. Such events will comprise the main data to enable a comparison of the treatment arms.

In summary, this pilot study draws together data from animal experiments and early human trials to explore a relatively new stroke therapy paradigm, that is, an anti-haemorrhagic transformation strategy for stroke patients treated with tPA. This is a slightly different concept to the traditional idea of trial of a neuroprotective agent in stroke. The pragmatic trial design and affordability of the study drug make this a relatively simple clinical study that could be conducted in most stroke units that offer thrombolytic therapy. Minocycline has practical advantages, including its well-established track record of safety (when used as an antibiotic), bioavailability (including orally), and the fact that it can be safely used in patients with haemorrhagic stroke. This makes it a candidate for use in the prehospital setting, without the need for imaging prior to administration. It is ease of use and inexpensiveness also make it a candidate for widespread use without concomitant thrombolytic therapy in nontertiary, rural, and even third world settings [18].

## Figures and Tables

**Table 1:** Sample size calculations table according to different treatment effects.

n for a given prevalence and reduction	Power = 75%		Power = 80%		Power = 85%		Power = 90%	
	25%	50%	25%	50%	25%	50%	25%	50%
0.04	3385	583	3891	670	4524	779	5389	928
0.06	2305	397	2649	456	3080	531	3669	632
0.08	1766	304	2030	350	2361	407	2812	484
0.10	1444	249	1660	286	1930	333	2299	396
0.20	812	140	934	161	1086	187	1293	223
0.30	619	107	711	123	827	143	985	170
0.40	542	93	623	107	724	125	862	149
